# A comprehensive review of COVID-19 characteristics

**DOI:** 10.1186/s12575-020-00128-2

**Published:** 2020-08-04

**Authors:** Hanie Esakandari, Mohsen Nabi-Afjadi, Javad Fakkari-Afjadi, Navid Farahmandian, Seyed-Mohsen Miresmaeili, Elham Bahreini

**Affiliations:** 1 Department of Biology, Science and research branch, Islamic Azad University of Tehran, Tehran, Iran; 2 Department of Biochemistry, Faculty of biological science, Tarbiat Modares University, Tehran, Iran; 3 Department of biology, Ashkezar branch, Islamic Azad University of Yazd, Ashkezar, Yazd Iran; 4 Department of Biochemistry, Faculty of Medicine, Iran University of Medical Sciences, P.O. Box: 1449614525, Tehran, Iran; 5 Department of Biology, Faculty of Sciences, Yazd University, Yazd, Iran

**Keywords:** Coronavirus, COVID-19, SARS-CoV-2, 2019-nCoV, Viruses, Epidemic disease

## Abstract

In December 2019, a novel coronavirus, named Severe Acute Respiratory Syndrome Coronavirus 2 (SARS-CoV-2) or (2019-nCoV) with unknown origin spread in Hubei province of China. The epidemic disease caused by SARS-CoV-2 called coronavirus disease-19 (COVID-19). The presence of COVID-19 was manifested by several symptoms, ranging from asymptomatic/mild symptoms to severe illness and death. The viral infection expanded internationally and WHO announced a Public Health Emergency of International Concern. To quickly diagnose and control such a highly infectious disease, suspicious individuals were isolated and diagnostic/treatment procedures were developed through patients’ epidemiological and clinical data. Early in the COVID-19 outbreak, WHO invited hundreds of researchers from around the world to develop a rapid quality diagnosis, treatment and vaccines, but so far no specific antiviral treatment or vaccine has been approved by the FDA. At present, COVID-19 is managed by available antiviral drugs to improve the symptoms, and in severe cases, supportive care including oxygen and mechanical ventilation is used for infected patients. However, due to the worldwide spread of the virus, COVID-19 has become a serious concern in the medical community. According to the current data of WHO, the number of infected and dead cases has increased to 8,708,008 and 461,715, respectively (Dec 2019 –June 2020). Given the high mortality rate and economic damage to various communities to date, great efforts must be made to produce successful drugs and vaccines against 2019-nCoV infection. For this reason, first of all, the characteristics of the virus, its pathogenicity, and its infectious pathways must be well known. Thus, the main purpose of this review is to provide an overview of this epidemic disease based on the current evidence.

## Background

In December 2019, an *outbreak of pneumonia* with unknown origin began in China’s Hubei Province, raising global health concerns due to the ease of transmission. To quickly diagnose and control the highly infectious disease, suspected people were isolated and diagnostic/ therapeutic procedures were developed via patients’ epidemiological and clinical data. After numerous studies, a novel severe acute respiratory syndrome coronavirus 2 (SARS-CoV-2) was identified as the cause of the disease, and the disease was dubbed “coronavirus-19″ (COVID-19) by Chinese Scientists [1, 2]. The presence of COVID-19 is manifested by several symptoms, ranging from asymptomatic/mild symptoms to severe illness and death. Common symptoms include cough, fever, and shortness of breath. Other reported symptoms are weakness, malaise, respiratory distress, muscle pain, sore throat, loss of taste and/or smell [3].

Clinical diagnosis of COVID-19 is based on clinical manifestations, molecular diagnostics of the viral genome by RT-PCR, chest x-ray or CT scan, and serology blood tests. The most common laboratory abnormalities in patients with positive RT-PCR are lymphopenia, leukopenia, thrombocytopenia, elevated CRP and inflammatory markers, elevated cardiac biomarkers, decreased albumin, and abnormal renal and liver function [4, 5]. However, several parameters may interfere with the results; the most important of which is the window period (time from exposure to the development of symptoms). As the body requires time to respond to the antigenic viral attack, symptoms may appear 2 to 14 days after exposure to the virus. The window-period of viral replication leads to false-negative results and problems in preventing COVID-19 expansion.

There have been two types of tests for COVID-19 during this pandemic: One type is PCR tests, as a molecular diagnostic technique based on viral genetic material that can diagnose an active COVID-19 infection. The early detection of COVID-19 via PCR depends on the presence of a sufficient amount of viral genome in the patient sample [6, 7] and the sensitivity of the RT-PCR assay. So, optimized or screening methods that able to detect the 2019-nCoV even in low viral titers are fairly necessary. The other type is serological tests based on antibodies against viral proteins. Serological tests identify people who have developed an adaptive immune response to the virus, as part of an active/or prior infection. Three types of antibodies including IgG, IgM, and IgA may be detected in response to the virus, especially IgM which is produced early after the infection [8]. It seems that serological tests, along with PCR increase the sensitivity/accuracy of the diagnosis, but due to window-period, immune tests do not help diagnose and screen in early infection. After infection with 2019-nCoV, it takes 2 weeks or more for antibodies to be detected [9]. Thus, early IgM/IgG antibody tests cannot detect active viral shedding in early infection, and if an individual is infectious. In other words, due to the direct identification of viral RNA, molecular tests are more sensitive than immune and serological tests in the diagnose of primary infection and can accelerate early screening even during the incubation period of COVID-19 (before symptom onset). So, immune tests will be practical and necessary for the event of a second recurrence of the virus in the society. Chinese researchers have reported a variety of results related to immune response, such as a broad range of antibodies between people with mild symptoms of the virus, while fewer antibodies among younger people, and no trace of antibodies in some individuals [10]. Thus the question arises as to whether a person with a positive RT-PCR test and severe, mild, or asymptomatic infection may still be prone to a second infection.

### Coronavirus Virology

**Fig. 1:**
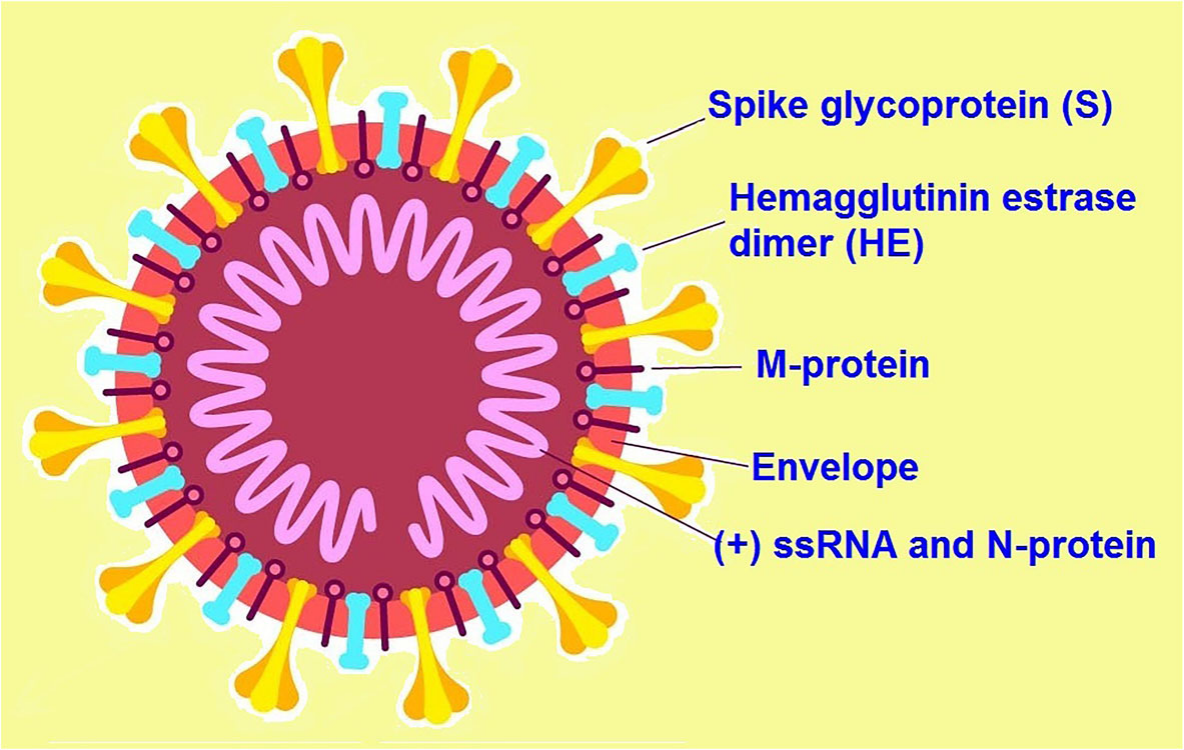
Typical structure of 2019-nCoV

A human coronavirus was isolated for the first time from the nasal secretions of a male child with a common cold in 1965 by Tyrell and Bynoe [11]. Because of their morphological similarity with a solar corona under an electron microscope (crown-like), the virus was termed coronavirus. Such appearance is because of the spike [S] glycoprotein radiating from the viral surface virus (Fig. 1). The S glycoprotein and the transmembrane glycoprotein [M] are two major envelope proteins. The S glycoprotein is an antigen that binds to the receptor and is responsible for cellular fusion. M glycoprotein has a role in envelope formation and virion assembly [10, 12]. The positive single-stranded RNA genome with about 26–32 Kbp, is the largest genomic RNA known among viruses and contains 7–10 different open reading frames. It is methylated in 5′ and has a poly-A tail in 3′. Genomic RNA is associated with the capsid by the basic phosphoprotein [N] [13, 14].

The Coronaviruses have four subfamilies including alpha, beta, gamma, and delta. The alpha and beta coronaviruses originate from mammals, while gamma and delta coronaviruses have been identified in pigs and birds. Beta-coronaviruses are also called bat-coronavirus. Bioinformatics analysis shows that RNA sequence of 2019-nCoV is more than 90% similar to a bat-coronavirus RaTG13. It has been reported that the beta-coronaviruses cause severe disease while the alpha-coronaviruses cause asymptomatic or mildly symptomatic disease [15, 16]. 2019-nCoV is closely related to the B lineage of the beta-coronaviruses, which are known to cause severe disease and fatalities [17]. To determine amino acid sequence and structure of 2019-nCoV proteins and subsequently to predict their interactions to host cells, the complete virus genome was sequenced and also was deposited in NCBI (GenBank: MN908947.3). Studies have revealed the outer membrane spike glycoprotein as the prime viral adhesion factor interacts with host cell targets such as ACE2, Ezrin, CD26, cyclophilins, and other cell adhesion factors [18, 19].

### Comparison of the Coronaviruses

**Fig. 2:**
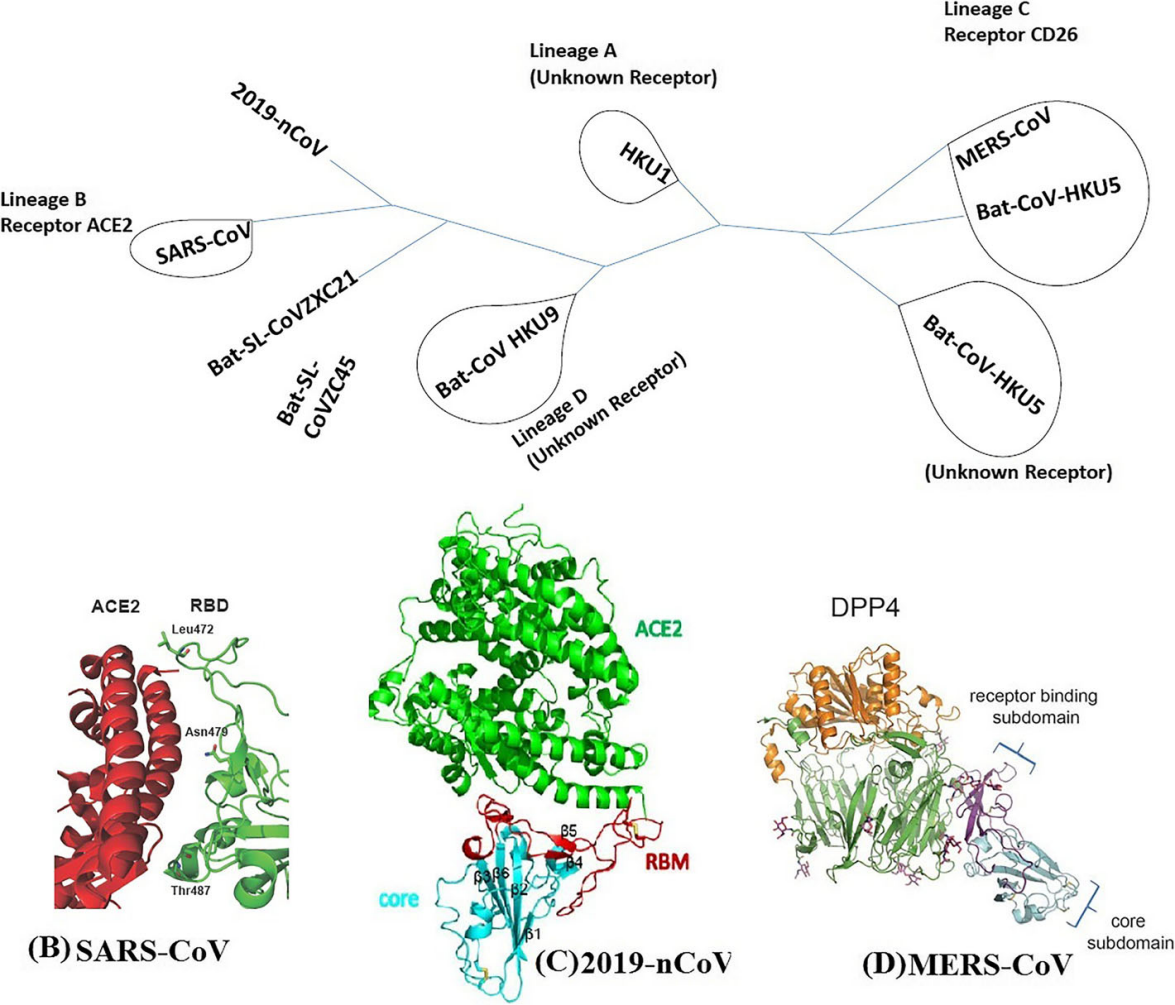
The phylogenetic illustration of the receptor-binding domain (RBD) in various betacoronaviruses **a**. The structure of RBD in SARS-CoV **b**, 2019-nCoV **c**, and MERS-CoV **d** [21]

The recent COVID-19 outbreak may be comparable with the outbreaks of the SARS-CoV (2002–2003) in China and with the Middle East Respiratory Syndrome Coronavirus (2012) in Saudi Arabia for their zoonotic transmission and some similarities in clinical features [20]. However, phylogenetic analysis (Fig. 2) of the receptor-binding domain (RBD) of betacoronavirus lineages indicates that 2019-nCoV closely belongs to two bat-derived SARS-like coronaviruses (bat-SL-CoVZC45 and bat- SL-CoVZXC21) with 88–89% similarity, whereas its similarity to the SARS-CoV and MERS-CoV is 50 and 79%, respectively [22]. It is worth noting that although there are significant genetic differences between these coronaviruses and the subgroup with 2019-nCoV, cross-reactions in RT-PCR or antibody measurements for SARS or other beta-coronaviruses my occur, if the primers and antigenic epitopes are not carefully selected [23, 24].

RBD located at the C-terminal domain of the spike protein mostly attaches to angiotensin-converting enzyme 2 (ACE2) located in the host cell membrane. ACE2 is mainly distributed in the epithelial cells of the lung and gastrointestinal tract. So, severe infection may occur in tissues with high expression levels of ACE2 including lung, intestine, kidney, and blood vessels [25].

Using the Swiss-Model program 33, the three-dimensional structure of the 2019-nCoV-RBD (Protein Data Bank ID: 2DD8), like the RBD in other beta-coronaviruses, consists of a core and an external subdomain (Fig. 2b-d). Interestingly, the similarity among the external subdomain of RBD in 2019-nCoV with that in SARS-CoV suggests the 2019-nCoV also uses ACE2 binding to entre into the host cell [25]. Moreover, the modeling studies have disclosed several RBD residues responsible for the binding of the 2019-nCoV to the ACE2 receptor, such as Asn439, Asn501, Gln493, Gly485, and Phe486 that differ from those in the SARS-CoV-RBD [6, 26].

### Pathogenesis of the 2019-nCoV

Acute 2019-nCoV infections are very similar to seasonal flu with the most common symptoms of fever, headache, shortness of breath, cough, muscle aches, and tiredness [1, 27]. The severity of the disease in most infected people is mild to moderate, and they can manage their symptoms at home without the need for hospitalization. While patients with serious symptoms such as difficulty breathing, chest pain or pressure, and loss of speech or movement need urgent medical attention. Other disorders seen in acute conditions include hemoptysis, diarrhea, dyspnea, acute heart injuries, and ground-glass opacities.

The lungs are the primary site of 2019-nCoV infection. The chest CT of the infected patients usually shows bilateral ground-glass opacity lesions in the posterior and peripheral lungs that are reported as the characteristic of 2019-nCoV pneumonia [28]. Pathologic studies on biopsy samples of lung, liver, and heart obtained from death COVID-19 patients have revealed that the lung is the main affected tissue with pathological changes including hyperplasia of type II pneumocytes, damage to the alveolar epithelial cells, the formation of the hyaline membrane and diffuse alveolar damage [29]. Thrombotic microangiopathy, significant accumulations of CD4+ mononuclear cells around small thrombotic vessels, and notable hemorrhage appear to be important causes of death in these individuals. Activated local megakaryocytes in the lung, platelet aggregation, fibrin deposition, and clot formation are involved with the mentioned process [30]. Besides, the abundance of viral RNA in neutrophils within the alveoli and the existence of some degenerated neutrophils indicate the viral infection in these cells [31]. Megakaryocyte response and platelet production have also been reported in H1N1 influenza infections [32]. Multifocal hepatic necrosis, mild lymphocytic infiltration, sinusoidal dilation, and steatosis are pathologic changes observed in the liver of COVID-19 patients with moderate to severe illness [33]. Mild myocardial hypertrophy changes and focal fibrosis are tissue changes seen in the heart biopsies of death COVID-19 patients [29]. Therefore, the researchers believe that effective therapy for COVID-19 should not be limited only to the viral pathogen as a target, but also the microangiopathic and thrombotic effects of the virus, and body immune response to viral infection must be considered in the disease management [34, 35].

The human angiotensin-converting enzyme 2 (ACE2), known as the major receptor for the viral S protein provides the entry point for 2019-nCoV to capture and infect a wide range of human cells. DC-SIGN (CD209), CD147, and L-SIGN (CD209L) are also other entry receptors for 2019-nCoV. Thus, drugs that interfere with the interactions of the spike protein/ACE2, CD147, DC-SIGN or L-SIGN or with their gene expression may inhibit viral invasion.

ACE2 is found in many types of cells and tissues, including the lungs, blood vessels, heart, liver, kidneys, and gastrointestinal tract. It is also present in the epithelium lining the lung, the nose, and mouth [36]. It is highly abundant in type 2 pneumocytes, the important cells located in alveoli, where oxygen exchanged with carbon dioxide [37]. Regulating blood pressure and inflammation are the main functions of local ACE2 via the conversion of angiotensin II (Ang II) to other molecules that neutralize the effects of Ang II. It competes with ACE (Angiotensin-converting enzyme) in hydrolyzing inactive decapeptide Ang I. ACE2 hydrolyzes Ang I into the nonapeptide Ang(1–9) and decreases the available Ang I to be converted to AngII by ACE. It also hydrolyzes Ang II and Ang(1–9) into Ang(1–7). Unlike Ang II, Ang(1–7) is a vasodilator with anti-inflammatory effects acting through Mas receptors [38]. Thus, ACE2 is a negative regulator of local RAS in lung and other tissues. Occupying the ACE2 receptor by SARS-CoV-2 prevents it from performing its normal function, and breaking the Ang I and Ang II peptides. Naturally, there is a high concentration of ACE in the lung tissue. Thus, in ACE2 deficiency, ACE will be more active due to more available Ang I which is changed into Ang II. Increased local Ang II levels damage blood vessel linings and cause inflammation and tissue injury. For this reason, it is claimed that the renin-angiotensin- system has a serious role in COVID-19 pathogenesis [39]. So it can be claimed that the main destructive factor in the patients with severe COVID-19 is abnormal and high activity of local Ang II. Drugs that inhibit ACE or ACE inhibitors (ACEI) such as ramipril, lisinopril, and enalapril may prevent the injuries caused by Ang II via inhibiting its production without blocking the actions of ACE2.

In addition to ACE2, there are other enzymes capable to hydrolyze Ang-I or Ang (1–9) to Ang (1–7) such as Neprilysin, Prolylcarboxypeptidase, and Prolylendopeptidase. It appears that if the activity of these enzymes is up-regulated in the lungs of people with COVID-19, the effects of reduced ACE2 may be compensated. Among the mentioned enzymes, higher expression levels of Neprilysin have been detected in lung tissue, especially in the membrane of pulmonary epithelial cells [40]. In addition to the negative effect on Ang II production, it cleaves and inactivates some other vasoactive peptides such as substance P, and endothelin [41]. It degrades and inactivates bradykinin. Bradykinin is identified as a potent vasodilator and lowers blood pressure, but causes contraction in the non-vascular smooth muscle of the bronchi and intestines and may play a role in the pain mechanism [42]. So, Neprilysin can be considered as a potential target to control the severity of COVID-19 disease.

Both Prolylcarboxypeptidase and Prolylendopeptidase are lysosomal and cytosolic peptidase, respectively, that have been mainly expressed in white blood cells [43]. They have also been detected in lung, liver, and kidney tissues. In addition to their role in the destruction or maturation of a variety of peptides, both enzymes may be considered as protective agents against AngII-induced injuries due to the conversion of AngII to Ang(1–7). Prolylcarboxypeptidase also named angiotensinase C, activates bradykinin, and hydrolyzes plasma prekallikrein to active kallikrein [44]. However, some studies have reported an inflammatory role for Prolylcarboxypeptidase in the lungs [45] and other tissues [46, 47].

### Transmission of the 2019-nCoV

*Rhinolophus affinis* bat was introduced as the natural host of 2019-nCoV due to 96.2% of whole-genome similarity with the BatCoV-RaTG13 genome [48]. Evidence indicates that the transmission of SARS-CoV and MERS-CoV from an animal to a human requires an intermediate host such as palm civets for SARS-CoV and camels for MERS-CoV. Many researchers believe that due to similarities between SARS-CoV and the 2019-nCoV, another animal as an intermediate host is probably needed to transmit 2019-nCoV to humans. If true, finding the intermediate 2019-nCoV host is vital to prevent interspecies transmission. In this regard, pangolins have been suggested as an intermediate host for 2019-nCoV, however, this assumption has not yet been proven [49, 50].

According to WHO, transmissions are classified as scattered, clustered, and community-based. Scattered cases refer to a small number of cases that are identified locally. Local transmission shows the locations that have been reported as a source of infection. A cluster of cases refers to cases that are clustered in time, geographic location, and/or by a common exposure. Community transmission refers to the region experiencing higher outbreaks of local transmission. It can be characterized by a situation that the source of exposure cannot be found or by a large number of cases that are not linkable to transmission chains and are identified through surveillance of a specific group of people [51, 52].

The routes of human-to-human transmission of 2019-nCoV among individuals include direct inhalation of contaminated droplets released into the environment by sneezing or coughing, and contact transmission via oral, nasal, and eye mucous [53]. Although a 6-ft distance is emphasized to protect against the spread of the disease, it is not enough. Microbes in droplets < 5 μm in diameter can stay in the air for a long time and can be transmitted to others over distances of more than 1 m [54]. Dental procedures are also a high-risk transmission route due to face-to-face communication and the presence of contamination with saliva, blood, and other body fluids, as well as the use of sharp tools [55]. Transmission may also happen through objects and personal items in the near environment around the infected person. Therefore, COVID-19 can be transmitted through direct contact with infected people or indirectly, through the surfaces or objects contaminated by an infected person [56]. Positive COVID-19 patients extensively contaminate their bedrooms, toilets and bathrooms; therefore, daily disinfection of their living environment, high touch surfaces, bathtubs and toilet bowls is essential. Water places such as swimming pools, rivers, lakes and ponds are also places that may be exposed to contamination by positive COVID-19 people. There is no evidence that 2019-nCoV is spread through water in pools, rivers, lakes [57]. To date, no reports of positive crowns have been received from water play places [58]; however, it cannot be said that it is completely 100% safe.

Intestinal infection and the presence of 2019-nCoV in feces have been reported, but there is not enough evidence for fecal-oral transmission of 2019-nCoV. Song et al. examined the presence of 2019-nCoV in testicular biopsy and semen of COVID-19 patients and did not find positive RT-PCR [59]. They stated that 2019-nCov does not infect the testes and the virus may not be sexually transmitted by infected men. Some studies have shown the presence of asymptomatic viral carriers with normal laboratory and chest CT findings [60, 61]. The mechanism by which asymptomatic carriers can obtain and transmit 2019-nCoV requires further study. Thus, an effective intervention is needed to prevent and control the spread of 2019-nCoV.

### Effect of COVID-19 on Other Organs

**Fig. 3:**
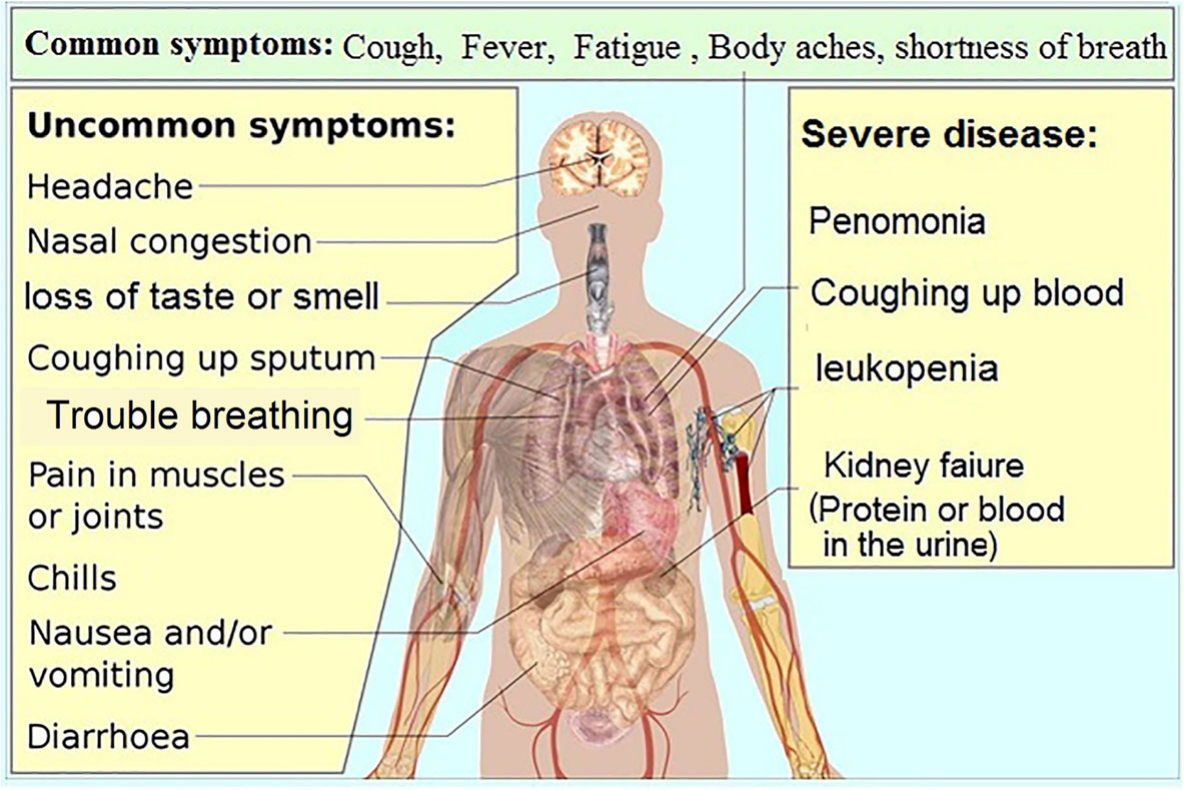
The systemic disorders caused by the COVID-19

In addition to respiratory illnesses, which may be associated with pneumonia, sepsis, or lung failure, evidence suggests that COVID-19 may affect other parts of the body as well [62]. Figure 3 illustrates some common, uncommon, and severe symptoms in patients with COVID-19.

According to several studies, 2019-nCoV infection, similar to some viral infections, may be accompanied by cardiac injury. A study of 400 patients hospitalized in Wuhan, China, found that about one-fifth of patients with COVID-19 developed heart disease, which increased the mortality rate in patients [63]. Severe and sudden inflammation of the heart muscle causes arrhythmias and impairs the heart’s ability to efficiently pump blood [64]. Therefore, patients with a history of cardiovascular disease and with high blood pressure are at higher risk of death than normal individuals. Oxygen deficiency due to trauma in the lungs damages the lining of the heart and blood vessels [65]. Besides, fatty plaques in the arteries of the heart of people with or without symptoms of cardiovascular disease may become unstable due to fever and inflammation, leading to vascular obstruction and cardiovascular problems [66]. Other possible disorders seen in hospitalized patients with COVID-19 are abnormal blood clotting and venous thromboembolism, which necessitate the administration of anticoagulants or thromboprophylaxis for these patients [67]. The secretion of various types of inflammatory cytokines in these conditions can exacerbate these complications [2]. Thus, cytokine inhibitors may be effective in reducing the severity of the disease.

Some studies have reported that COVID-19 may damage CNS. Some observed symptoms include losing the senses of smell, taste or vision, and decreasing alertness [68]. Also, seizures, stroke, and acute necrotizing hemorrhagic encephalopathy have been reported in patients with severe COVID-19 infection [69]. Therapeutic results have shown that neurological symptoms gradually decrease in patients receiving viral encephalitis treatment [70].

About half of patients with COVID-19 show evidence of protein or blood in the urine, which indicates early renal damage [71]. It has been reported that 15 to 30% of hospitalized patients with COVID-19 in China and New York need to receive renal treatments or dialysis [72, 73]. However, the direct attack of the virus on the kidneys is still being debated.

The presence of the virus in the fecal samples of some patients with COVID-19 indicates that the virus can reach the human gastrointestinal tract. About half of all patients suffer from vomiting, diarrhea, and other gastrointestinal disorders. Acute viral hepatitis has also been found in some of these patients [74, 75]. After developing symptoms of fever and cough, physicians connect gastrointestinal disorders with COVID-19.

### Treatment of the COVID-19

Most viral infections target a series of “protective responses” in the host body, including apoptosis, stress response, autophagy, and innate immunity. The strength of the body’s protective response depends on genetics, epigenetics, and or other factors, such as lifestyle [76]. The results of epidemiological and clinical studies show that most of the infected individuals who are asymptomatic or show mild symptoms have good body capacity for protective responses to activate the body’s antiviral defense mechanisms including immune cell defense and interferons induction. But, such supportive and immune responses are weak in most elderly people or patients with immunodeficiency, lung problems such as fibrosis, chronic obstruction and asthma, cardiovascular and hypertension problems, diabetics, or obesity. Such infected people may encounter more severe symptoms of the disease, severe respiratory problems, and even death [76–78]. Weak immune responses and inability to fight the virus increase the viral load, leading to increased secretion of inflammatory cytokine in the bronchoalveolar lavage fluid and severe inflammatory/oxidative stress response, followed by severe lung damage. Considering acute respiratory distress syndrome, antiviral therapy, antibiotics, corticosteroids, and anti-inflammatory drugs are commonly used in treatment protocols [79].

Most drug designs against 2019-nCoV have been focused on immunomodulators (such as corticosteroids and interferons), monoclonal antibody production, and inhibitory agents against viral proteinase, helicase, and polymerases [80]. However, to date, no specific antiviral treatment or vaccine has been approved by the FDA for COVID-19. Due to short replication times and high viral yields, the replication of the positive-sense viral RNA genome undergoes high error rates and homologous and nonhomologous recombination. This genetic plasticity makes it difficult to design an anti-COVID-19 drug and vaccine [81, 82].

Clinical control of the disease is only based on symptoms via available therapeutic drugs, and in severe cases, via supportive care including oxygen and mechanical ventilation. The available therapeutic agents used for the treatment of COVID-19 patients are antiviral agents including Remdesivir, Chloroquine, Tocilizumab, Hydroxychloroquine, Umifenovir, Lopinavir, Oseltamivir, and Favipiravir, and adjunctive agents such as zinc, vitamin D, Azithromycin, Ascorbic acid, Nitric oxide, Corticosteroids, IL-6 antagonists. Because the viral load reaches its peak around the time of symptom onset, the combination of multiple antiviral drugs, along with adjunctive agents may quickly suppress the amount of virus in a patient’s body and may be effective in reducing the severity of the disease and the duration of the viral infection [83]. Vitamin D as an adjunctive agent can reduce the risk of viral infections and prevent pneumonia and lung damage through a variety of mechanisms, including reducing the level of pro-inflammatory cytokines and increasing the concentration of anti-inflammatory cytokines [84]. Thus, Vit D may be effective in reducing the severity of COVID-19 disease. Zinc is another adjunctive agent that is known for its antiviral, antibacterial, and anti-inflammatory properties. It is involved in a variety of mechanisms, including inhibition of NF-κB signaling, regulation of T-cell, and restriction of cytokine storms [85]. Speth et al. in their animal study reported that zinc reduced the activity of recombinant human ACE-2 in rat lungs [86].

The mentioned antiviral drugs are not specific to 2019-nCoV and may have limitations in use for treatment. For example, Actemra (tocilizumab) is a monoclonal antibody against the recombinant human IL-6 receptor from the IgG1τ subclass. At first, it was used to treat rheumatoid arthritis and juvenile idiopathic arthritis. Then, it was administered against cytokine storms in coronavirus and influenza infections that are characterized by high levels of IL-6 and other cytokines. Actemra successfully could diminish the cytokine storm associated with viral infections. Tian et al. in their research proposed that due to high similarity among receptor-binding domains in 2019-nCoV and SARS-CoV, SARS-CoV-specific human monoclonal antibody, CR3022, might also interact with 2019-nCoV spike protein and might be used as vaccines against COVID-19 disease, but CR3022 failed to bind 2019-nCoV spike protein. Thus, more research is needed to develop novel monoclonal antibodies that can be specifically linked to 2019-nCoV RBD [87]. Other examples are Chloroquine and Hydroxychloroquine, that have been used as antimalaria for more than 70 years and also as anti-amoebiosis and anti-human immunodeficiency virus. In-vitro and small clinical studies have shown that both drugs could be effective against SARS-CoV infection and prevent from the virus spread [88], but several clinical studies have reported that administration of chloroquine and hydroxychloroquine is associated with an increased risk of heart problems in COVID-19 patients, including cardiac arrhythmias and cardiac arrest [89].

In addition to chemical drugs, traditional herbal medicines and convalescent plasma have also been suggested to treat COVID-19. WHO believes that traditional, complementary, and alternative medicine has many benefits. Some countries including Iran, China, India, Korea, and parts of Africa have a long history of traditional medicine and have suggested medicinal guidelines for treating COVID-19. For example, the Chinese have reported that than 85% of COVID-19 patients in China had received Traditional Chinese Medicine treatments. Due to the similarity among SARS-CoV and 2019-nCoV in the pathogenesis via using *receptor ACE2* for *host* cell entry, the same herbal medicines traditionally used for SARS-CoV were also used for 2019-nCoV in China and Korea. Some of the commonly used herbal medicine in China include *Astragalus Membranaceus*, *Saposhnikoviae Divaricata*, *Glycyrrhiza Uralensis*, *Lonicerae Japonicae Flos*, *Rhizoma*, *Atractylodis Macrocephalae*, *Atractylodis Rhizoma*, and *Fructus forsythia*. Some herbal products such as *Re Du Ning* and *Shen Fu* have immunosuppressive effects via a decrease in the level of IL-1β, IL-6, IL-8, IL-10, TNF-α, and other cytokines. The herbal formula of *Qingfei Paidu* can regulate the immune-related pathway and reduce inflammation in lung and spleen of patients.

Ayurveda, Siddha, Unani, and Homeopathy (referred to as AYUSH) are Indian medicinal systems that use natural drugs of plant, animal, and mineral origin for treatment. At present pandemic, AYUSH has recommended that the Homeopathy and Ayurveda as immune-boosters have sufficient potential to prevent and treat COVID-19. Considering the success of AYUSH systems in managing several epidemics and restoring health, AYUSH system recommends some herbs including *Chyavanprash*, *Herbal tea*, and *Turmeric milk* as immune boosters and has suggested some herbal formulations for treating COVID-19 [90, 91].

In Iran, an herbal formula called *Imam Kazem* has been reported by Islamic medicine, which is said to be effective in treating colds and influenza. This herbal formula was used for COVID-19 patients and they claimed that it could prevent the disease from getting worse and reduce the symptoms of the disease. The composition of this herbal formula in summer is *Terminalia chebula*, *Foeniculum vulgare*, and *red sugar*, and in winter it consists of *Terminalia chebula*, *Pistacia lentiscus* and *red sugar* (*Red sugar* means sugarcane sugar that has not been processed industrially). Despite the positive reports of this herbal formula in the treatment of lung diseases in our country, no experimental or clinical reports were found to refer. *Rosewater* as other herbal products is flavored water made by steeping rose petals in water. In addition to oral consumption, it has antimicrobial properties and can be sprayed on surfaces and space as a disinfectant solution [92].

Other examples of herbal medicine used in COVID-19 treatment include: *Ginseng* (*Panax ginseng*) regulates the activity of immune cells including T cells, and B cells, macrophages, dendritic cells, natural killer cells [93]; *Ginger* (*Zingiber officinale*) has anti-apoptotic, anti-inflammatory, anti-tumor activities, anti-tumourigenic, anti-hyperglycaemic, antioxidant, and analgesic properties [94]; *garlic* (*Allium sativum*) product is a strong immune stimulator [95]; *Echinacea* extract (*Echinacea purpurea (L.) Moench*) with antimicrobial and antioxidant activities is used to improve the immune system and to treat pulmonary symptoms caused by bacterial infections [96].

Despite such treatments, there are few reports of improved patients with negative COVID-19 test, that after a while, their COVID-19 test has been positive again. The hospital reports have confirmed, it is possible that improved patients with negative COVID-19 tests become positive again for 2019-nCoV RNA, although a small portion of discharged COVID-19 has shown recurrent recurrences. There are reports of improved people that showed second recurrences with positive PCR tests after discharge from the hospital or during quarantine, and were hospitalized again. These people were mostly asymptomatic, but their blood IgM and IgG were still positive [97–99]. Due to the possibility of recurrence of the infection, all discharged patients should be quarantined for at least 14 days and tested regularly for 2019-nCoV RNA. During quarantine, they should avoid contact with others because they may still be a virus carrier and spread the infection to others.

## Conclusions

2019-nCoV exists in the respiratory, fecal, and blood samples of COVID-19 patients. However, the main route of transmission is through inhalation of respiratory droplets or contact with contaminated fomites due to the persistence of the virus on surfaces. Although many treatments have been proposed, there are currently no specific options for treating COVID-19 or preventing 2019-nCoV infection. Like influenza, the prevalence of COVID-19 is expected to decrease as the weather warms up in the summer and the 2019-nCoV infection is predicted to start again with the gradual cooling of the air in the fall. Unfortunately, it is not yet possible to say that improved people from COVID-19 are resistant or susceptible to the second infection. Due to the high rate of mortality and economic damage to various communities to date, a significant attempt should be made to produce successful drugs and vaccines against 2019-nCoV infection. However, in the absence of vaccines and antivirals, the most important way to control the disease among the populations is regular hand washing, the use of disinfectants, and the prevention of contact with the face and mouth after interacting with the infected environment.

## Data Availability

Data presented in this manuscript is available upon request.

## Abbreviations

COVID-19: Coronavirus disease-19
SARS-CoV-2: Severe acute respiratory syndrome coronavirus 2
2019-nCoV: Novel coronavirus
ACE: Angiotensin-converting enzyme
MERS-CoV: Middle East Respiratory Syndrome Coronavirus
RBD: Receptor-binding domain
ACE2: Angiotensin-converting enzyme 2
